# Metabolomics comparison of rumen fluid and milk in dairy cattle using proton nuclear magnetic resonance spectroscopy

**DOI:** 10.5713/ajas.20.0197

**Published:** 2020-06-24

**Authors:** Jun Sik Eom, Eun Tae Kim, Hyun Sang Kim, You Young Choi, Shin Ja Lee, Sang Suk Lee, Seon Ho Kim, Sung Sill Lee

**Affiliations:** 1 Division of Applied Life Science (BK21Four), Gyeongsang National University, Jinju 52828, Korea; 2 National Institute of Animal Science, Rural Development Administration, Cheonan 31000, Korea; 3 Institute of Agriculture and Life Science & University-Centered Labs, Gyeongsang National University, Jinju 52828, Korea; 4 Ruminant Nutrition and Anaerobe Laboratory, College of Bio-industry Science, Sunchon National University, Suncheon 57922, Korea

**Keywords:** Dairy Cattle, Metabolites, Milk, Rumen Fluid, ^1^H-NMR Spectroscopy

## Abstract

**Objective:**

The metabolites that constitute the rumen fluid and milk in dairy cattle were analyzed using proton nuclear magnetic resonance (^1^H-NMR) spectroscopy and compared with the results obtain for other dairy cattle herds worldwide. The aim was to provide basic dataset for facilitating research on metabolites in rumen fluid and milk.

**Methods:**

Six dairy cattle were used in this study. Rumen fluid was collected using a stomach tube, and milk was collected using a pipeline milking system. The metabolites were determined by ^1^H-NMR spectroscopy, and the obtained data were statistically analyzed by principal component analysis, partial least squares discriminant analysis, variable importance in projection scores, and metabolic pathway data using Metaboanalyst 4.0.

**Results:**

The total numbers of metabolites in rumen fluid and milk were measured to be 186 and 184, and quantified as 72 and 109, respectively. Organic acid and carbohydrate metabolites exhibited the highest concentrations in rumen fluid and milk, respectively. Some metabolites that have been associated with metabolic diseases (acidosis and ketosis) in cows were identified in rumen fluid, and metabolites associated with ketosis, somatic cell production, and coagulation properties were identified in milk.

**Conclusion:**

The metabolites measured in rumen fluid and milk could potentially be used to detect metabolic diseases and evaluate milk quality. The results could also be useful for metabolomic research on the biofluids of ruminants in Korea, while facilitating their metabolic research.

## INTRODUCTION

Metabolomic studies have been actively conducted to not only investigate metabolic diseases found in animals, but also related human diseases, drug toxicity, and altered gene functions [1–3]. Metabolomics is the study of metabolic mechanisms that are achieved by measuring metabolic products in biological specimens as a whole and by elucidating the underlying chemical processes [2]. The most common techniques used for metabolomics are nuclear magnetic resonance (NMR) spectroscopy, liquid chromatography-mass spectrometry (LC/MS) and gas chromatography-MS (GC/MS). NMR spectroscopy is less sensitive than LC/MS and GC/MS, while providing at relatively simple sample preparation and highly reproducible molecule quantification [3,4]. In addition, NMR is a powerful analytical technique for the identification of metabolic biomarkers [5]. Therefore, NMR would be suitable for the analysis of numerous metabolites in the biofluids of ruminants.

Numerous researches have previously analyzed the metabolites in ruminant biofluids (e.g., rumen fluid, serum, milk, and urine) using NMR spectroscopy, and by the identification and quantification of metabolites through concentration comparison [1,6–8]. Moreover, metabolic pathways have been statistically analyzed by principal component analysis (PCA), partial least square discriminant analysis (PLS-DA), determination of variable importance in projection (VIP) scores, and hierarchical clustering analysis (with heatmaps) [1,6–8]. In particular, research is being conducted on finding new biomarkers for metabolic diseases found in ruminants (acidosis, ketosis, etc.) as well as for the early diagnosis of these diseases that may occur in the livestock industry; this has been achieved by identifying specific metabolites in ruminant biofluids [9,10]. Therefore, metabolites in ruminant biofluids must be studied to prevent and minimize the damages caused by metabolic diseases.

In Korea, NMR metabolomics studies have been conducted on samples such as natural food (potatoes and *Astragalus* root) products [11,12], human urine [13], liver, serum, urine of piglets [14], and obese mice [15]. Recently, metabolomic research has been conducted by comparing the composition of rumen fluid in Hanwoo’s cattle with those of the volatile fatty acids and monosaccharides in metabolites by using NMR spectroscopy, high performance liquid chromatography and high-performance anion-exchange chromatography [16]. A study has also investigated the effects of different roughage diets that were fed at concentrated ratios on the milk productivity and milk metabolites in dairy cows [17]. However, extremely few studies have been conducted on metabolites that constitute ruminant biofluids in Korea by using proton nuclear magnetic resonance (^1^H-NMR).

Therefore, this study aimed to measure the metabolites in dairy cow rumen fluid and milk by using ^1^H-NMR spectroscopy; these metabolites were quantified and classified to construct a database for each sample, wherein the concentration of each metabolite was provided. The role of metabolites in rumen fluid and milk observed in this study were compared to that of the metabolites in ruminant biofluids reported in a previous study. Moreover, the results of this study provide a useful a database for the analysis of metabolites in ruminant biofluids.

## MATERIALS AND METHODS

All experimental protocols used in this study were approved by the National Institute of Animal Science Department of Animal Resources Development Dairy Science Division (Cheonan, Chungcheongnam-do, Korea; NIAS-201908).

### Animals and sample collection

Six dairy cattle were used in this study, and all animals were fed the same diet that was composed of total mixed ration (TMR); the fed amounts were defined by their voluntary intake. The chemical composition of the TMR have been presented in Table 1. Rumen fluid samples were collected using stomach tube from dairy cows. All rumen fluid collected using conical tube (30 mL each). Subsequently, rumen fluid samples centrifuged at 806 g for 15 min to remove feed particles and the supernatant was stored at −80°C until analyzed for metabolites using ^1^H-NMR. Milk samples were collected by using pipeline milking system and then transferred to conical tubes (30 mL each). Samples were stored at −80°C until analyzing for metabolites by ^1^H-NMR.

### NMR spectroscopy

The rumen fluid sample was recentrifuged at 12,902 g for 10 min and the supernatant was collected 300 μL. Standard buffer solution (2,2,3,3-d(4)-3-(trimethylsilyl)propionic acid [TSP] sodium salt) was added to 300 μL of supernatant in deuterium oxide (D_2_O) solvent/standard buffer solution (300 μL). The supernatants (600 μL) were transferred to 5 mm NMR tubes for NMR analysis [6].

The collected milk sample was centrifuged at 4,000 g for 15 min to remove the lipid layer in supernatant. Thereafter, the mixture of milk (250 μL) and D_2_O (300 μL) were transferred to 5 mm NMR tubes for NMR analysis [6].

^1^H-NMR spectra of rumen fluid and milk samples were obtained on a SPE-800 MHz NMR-MS Spectrometer (Bruker BioSpin AG, Billerica, MA, USA) at 64 K using a 5 mm triple-resonance inverse cryoprobe with Z-gradients (Bruker BioSpin CO., USA). The pulse sequence used for the rumen fluid and milk were a NOESY and Carr-Purcell-Meiboom-Gill pulse sequence collecting 64,000 data points with 128 transients, a spectral width of 16,025.641 Hz, a relaxation delay of 4.0 s, and an acquisition time of 2.0 s.

### NMR metabolite measurement, quantification, and statistical analysis

The processed spectra were imported into the Chenomx NMR suite 8.4 software (Chenomx, Edmonton, Canada) for identification and quantification. The baseline and phase were matched for comparison between samples using the NMR processor. The following procedure was employed for qualitative and quantitative analysis of the metabolites in samples: the spectral width was 10 ppm and was referenced to the TSP signal at 0 ppm. The resources used were the Livestock Metabolite Database (http://www.lmdb.ca), Bovine Metabolite Database (http://www.bmdb.ca), and Chenomx library. Moreover, metabolite qualitative and quantitative were performed using the Chenomx profiler program.

Statistical analyses of the metabolite data were conducted using MetaboAnalyst version 4.0 (http://www.metaboanalyst.ca), an open source R-based program for metabolomics. During the statistical analysis the resulting metabolites were subjected to sample normalization, data transformation, and data scaling by using the “sum”, “log”, “pareto” functions, respectively. Univariate Student’s t-tests were used to identify differences between the metabolite profiles of the rumen fluid and milk samples. PCA and PLS-DA were used as multivariate data analysis techniques to generate a classification model and provide quantitative information for discriminating the metabolites. The different rumen fluid and milk metabolites were determined on the basis of a statistically significant threshold of VIP scores. Metabolites with VIP scores higher than 1.5 were obtained through PLS-DA. Metabolic pathways were quantified and, common metabolites in rumen fluid as well as milk metabolites of the other studied animals were statistically analyzed by MetaboAnalyst 4.0 for performing a metabolic pathway analysis, based on a database source by Kyoto encyclopedia of genes and genomes (http://www.kegg.com).

## RESULTS

### Measured quantification of metabolites by ^1^H-NMR spectroscopy

**Figure 1:**
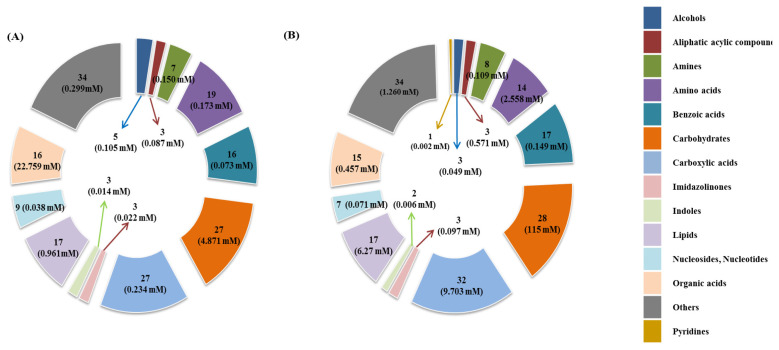
The classification of measured metabolites according to chemical class in rumen fluid (A) and, milk (B) by ^1^H-NMR analysis. Each square box color indicates the classification of metabolites, the numbers represents the measured metabolites, and the numbers in parentheses indicate the sum of the total concentrations of the measured metabolites. ^1^H-NMR, proton nuclear magnetic resonance.

The results in shown Figure 1 and Supplementary Table S1, S2, and S3 reveal the measured and quantified compound in rumen fluid (A) and milk (B) that were measured by ^1^H-NMR. In the rumen fluid, 186 metabolites were measured and classified into 13 chemical classes. The classes with the most metabolites were others (34), carboxylic acids (27), and carbohydrates (27); the classes with the three highest concentrations were organic acids (22.759 mM), carbohydrates (4.871 mM) and lipids (0.961 mM). In addition, 72 metabolites were quantified (n≥4) in the rumen fluid. In milk, 184 metabolites were measured and classified into 14 chemical classes. The classes with the most metabolites were others (34), carboxylic acids (32), and carbohydrates (28); the classes with the three highest concentrations were carbohydrates (115 mM), carboxylic acids (9.703 mM), and lipids (6.270 mM). In addition, 109 metabolites were quantified (n≥4) in milk.

### ^1^H-NMR spectroscopy analysis

**Figure 2:**
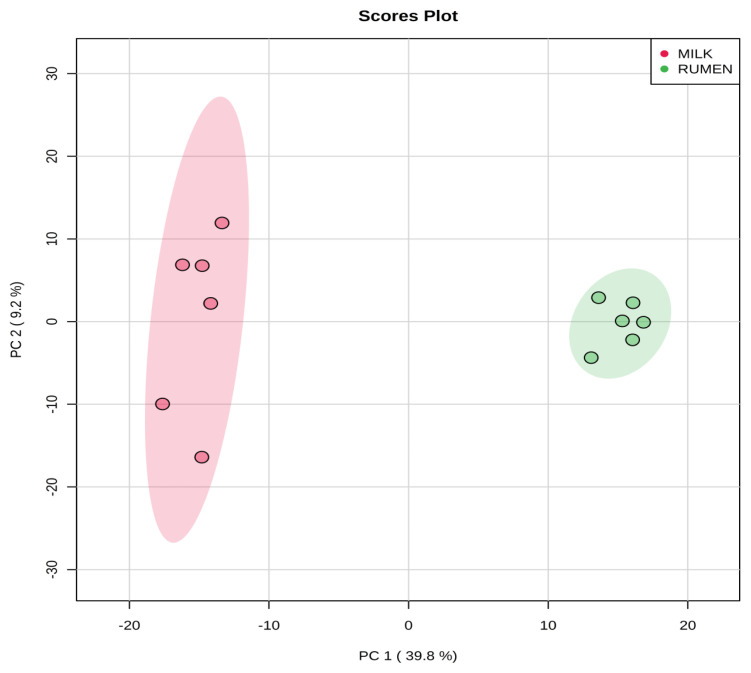
Principal components analysis (PCA) score plot based on metabolites data in rumen fluid and milk by ^1^H-NMR analysis. On the score plot, each point represents an individual sample, with the green dot representing the rumen fluid group (n = 6), and the red dot representing the milk group (n = 6). The abscissa and ordinate represent the variance associated with PC 1 and 2, respectively. ^1^H-NMR, proton nuclear magnetic resonance.

**Figure 3:**
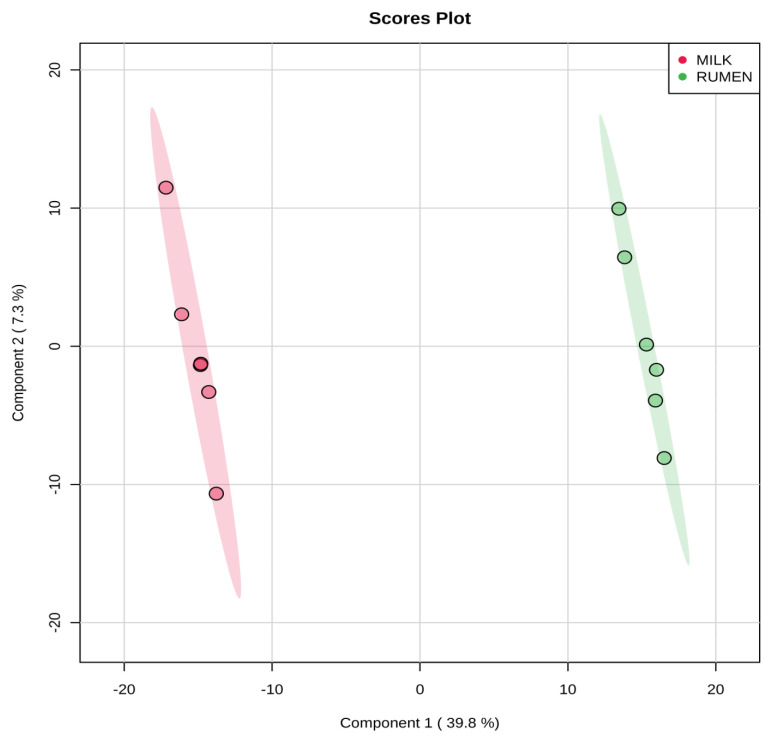
Partial least square discriminant analysis (PLS-DA) score plot of rumen fluid and milk, by ^1^H-NMR analysis. The shaded ellipses represent the 95% confidence interval estimated from the score. On the score plot, each point represents an individual sample, with the green dot representing the rumen fluid group (n = 6), and the red dot representing the milk group (n = 6). The abscissa and ordinate represent the variance associated with components 1 and 2, respectively. ^1^H-NMR, proton nuclear magnetic resonance.

**Figure 4:**
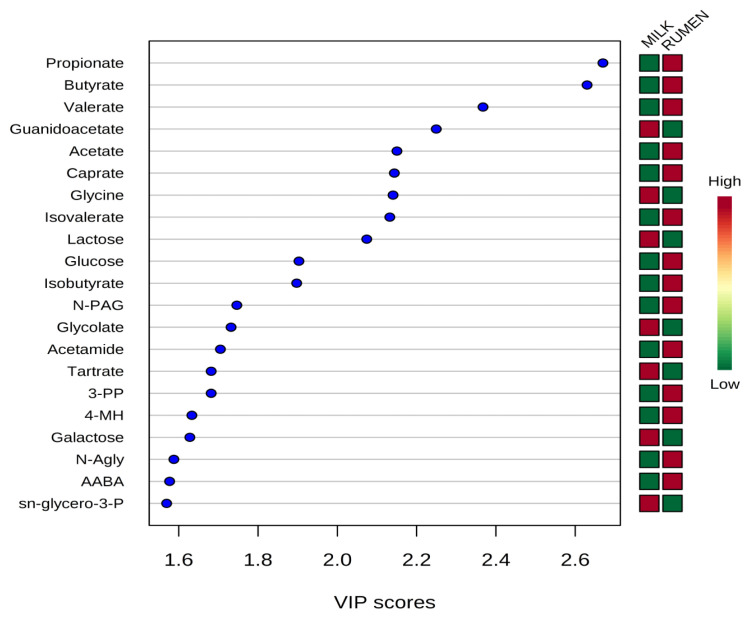
Variable importance in projection (VIP) scores of metabolites in rumen fluid and milk by ^1^H-NMR analysis. The selected metabolites were those with VIP score>1.5. Heat map with red or green boxes on the right indicates high and low abundance ratio, respectively, of the corresponding metabolite in rumen fluid and milk. VIP score was based on the PLS-DA model. ^1^H-NMR, proton nuclear magnetic resonance; PLS-DA, partial least square discriminant analysis; N-PAG, N-phenylacetylglycine; 3-PP, 3-phenylpropionate; 4-MH, 4-methylhistidine; N-agly, N-acetylglycine; AABA, 2-aminobutyrate; sn-glycero-3-P, sn-glycero-3-phosphocholine. VIP score value: propionate, 2.6698; butyrate, 2.6299; valerate, 2.3674; guanidoacetate, 2.2495; acetate, 2.1503; caprate, 2.1438; glycine, 2.1405; isovalerate, 2.1325; lactose, 2.0742; glucose, 1.9032; isobutyrate, 1.8975; N-PAG, 1.7465; glycolate, 1.7320; acetamide, 1.7051; tartrate, 1.6819; 3-PP, 1.6819; 4-MH, 1.6332; galactose, 1.6281; N-Agly, 1.5878; AABA, 1.5771; sn-glycero-3-P, 1.5696.

Representative ^1^H-NMR spectra of 58 and 63 metabolites measured as single, doublet, and triplet peaks, as well as the reference substance TSP, in the rumen fluid and milk samples are shown in Supplementary Figures S1 and S2, respectively. Our data showed that the metabolites were different between rumen fluid and milk. To visualize the differences among the metabolites data, we performed PCA and PLS-DA (Figures 2, 3). Both score plots revealed differences in rumen fluid and milk, which were well separated in PC1 (39.8%) and PC2 (9.2%) for PCA and PC1 (39.8%) and PC2 (7.3%) PLS-DA. These results indicated significant variation among the different classes and concentrations of metabolites in the rumen fluid and milk. As shown in Figure 4, the two biofluids exhibited completely different metabolite profiles. VIP scores were also utilized to identify the metabolites that affected the differentiation in the PLS-DA score plot, and 21 metabolites were found to be significantly different (VIP score >1.5) in the rumen fluid and milk (Figure 4). In the rumen fluid, 14 metabolites (propionate, butyrate, valerate, acetate, caprate, isovalerate, glucose, isobutyrate, N-phenylacetylglycine, acetamide, 3-phenylpropionate, 4-methylhistidine, N-acetylglycine, and 2-aminobutyrate) had significantly higher concentrations than those present in milk. In milk, 7 metabolites (guanidinoacetate, glycine, lactose, glycolate, tartrate, galactose, and sn-glycero-3-phosphocholine) had significantly higher concentrations than those present in the rumen fluid.

### Top 30 average concentrations of rumen fluid and milk metabolites

The top 30 average concentrations of metabolites in the rumen fluid and milk are shown in Table 2 and 3. Among the metabolites measured in the rumen fluid, acetate, propionate, and butyrate (classified as organic acids) had the highest concentrations. In contrast, uracil (classified as a nucleoside and nucleotide), 3-hydroxyphenylacetate (classified as a carboxylic acid), and imidazole (classified as an imidazolinone) had the lowest concentrations. Among the metabolites measured in milk, lactose (classified as a carbohydrate), guanidinoacetate (classified as a carboxylic acid), and ethylene glycol (classified as a lipid) had the highest concentrations. In contrast, tartrate (classified as a benzoic acid), N-carbamoyl-β-alanine (classified as an organic acid), and acetone (classified as a others compound) had the lowest concentrations.

### Common metabolites and metabolic pathways in the rumen fluid and milk

**Figure 5:**
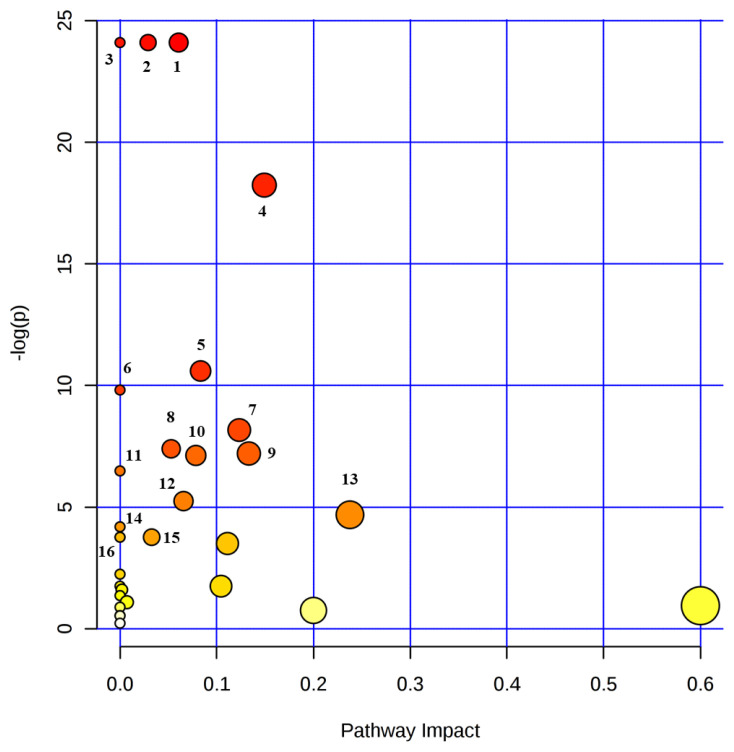
Metabolic pathway mapping of common quantified (n≥4) metabolites between rumen fluid and milk. The pathway impact analysis was performed using Metaboanalyst 4.0 software. The x-axis represents the pathway impact, and y-axis represents the pathway enrichment. The results are presented graphically as a bubble plot. The darker color and larger the size represent higher p-value from enrichment analysis and greater impact from the pathway topology analysis, respectively. Metabolic pathway name: 1, pyruvate metabolism; 2, glycolysis/glucoconeogenesis; 3, glyoxylate and dicarboxylate metabolism; 4, galactose metabolism; 5, glycerophospholipid metabolism; 6, ether lipid metabolism; 7, starch and sucrose metabolism; 8, tryptophan metabolism; 9, glycine, serine and threonine metabolism; 10, vitamin B_6_ metabolism; 11, arginine and proline metabolism; 12, amino sugar and nucleotide sugar metabolism; 13, histidine metabolism; 14, phenylalanine metabolism; 15, citrate cycle (tricarboxylic acid cycle); 16, propanoate metabolism.

Fourth-three common metabolites were quantified (n≥4) in the rumen fluid and milk (Supplementary Table S1–3). An analysis showed that the common metabolites (Table 4, Figure 5) followed 16 metabolic pathways, among which the top five pathways were pyruvate metabolism, glycolysis/gluconeogenesis, glyoxylate and dicarboxylate metabolism, galactose metabolism, and glycerophospholipid metabolism. The metabolites in the relevant pathways were mainly carbohydrates and amino acids.

## DISCUSSION

Metabolites in ruminant biofluids can be affected by the species of ruminant, type of feed consumed, breeding environment and season, and the adopted analytical technique. Saleem et al [18] analyzed a total of 246 metabolites using ^1^H-NMR, GC/MS, LCMS/MS, and ICP-MS to study bovine rumination according to feeding concentrate ratio, 50 of which were measured by 1H-NMR. In this study, a total of 186 metabolites were measured by ^1^H-NMR, 72 of which were quantified in rumen fluid. O’Callaghan et al [6] studied bovine milk under different types of irradiation type by ^1^H-NMR and quantified a total of 49 metabolites. In this study, a total of 184 metabolites were measured by ^1^H-NMR, among which 109 were quantified in milk. It is possible that the higher NMR frequency and the Chenomx programs used in this study resulted in the quantification of more metabolites.

PCA is a multivariate method that can be used to classify and analyze large amounts of data with numerous variables and provide information on metabolite changes [19]. PLS-DA visualizes metabolic changes that are likely to affect classification for two or more individuals. VIP scores and heatmaps indicate the differences in the contributions of metabolites when the two groups are divided according to PLS-DA [20]. In Korea, multivariate analysis has been widely used for food origin determination [11,12] and human diseases [13]. Multivariate analysis is also an essential method for metabolite research; thus, studies on the metabolites found in ruminant biofluids are also be necessary. The PCA of the metabolites in rumen fluid and milk showed a separation between the groups. The PLS-DA was influenced by propionate, butyrate, valerate in rumen fluid and guanidinoacetate, glycine, and lactose metabolites in milk, which also demonstrated a separation between the groups.

Previous studies have shown that acetate, propionate, and butyrate in the rumen account for approximately 70% of ruminant energy and the majority of metabolite concentrations [18], which in accordance with this study. In terms of metabolic diseases, a higher concentrate ratio of the agricultural feed can cause acidosis [21]. According to Ametaj et al [22], a diet with a high concentrate ratio increases methylamine, glucose, alanine, maltose, propionate, uracil, valerate, xanthine, ethanol, and phenylacetate concentrations and decreases 3-phenylpropionate concentrations. In this study, the feeding concentrate ratio was measured in relation to the metabolites. As reported by Wang et al [23], the concentrations of metabolites based on biogenic amines (tyramine, putrescine, histamine, methylamine, and tryptamine) in the rumen fluid were observed to be higher in bovines suffering from acidosis. Sato and Shiogama [24] reported that changes in the concentrations of acetone and isopropanol in rumen fluid might be an indicator of ketosis. Compared with the metabolites detected in the above-mentioned previous studies [22–24], a majority of these were also detected in the rumen fluid in this study, including the diuretic-related biogenic amine metabolite histamine, methylamine, acetone, and isopropanol. Therefore, the metabolites measured in rumen fluid in this study can be used to verify the occurrence of diseases such as acidosis and ketosis in dairy cattle.

Numerous studies have been conducted on metabolites present in milk. Among them, a few have determined the chemical composition and nutritional value of milk along with the bioactive compounds present in milk; further, they have elucidated, the potential use of biomarkers in milk as a diagnostic tool using NMR spectroscopy [25]. According to Klein et al [9], the milk samples from cows exhibiting ketosis showed higher concentrations of ketone body metabolites (3-hydroxybutyrate [BHBA], acetoacetate, and acetone) and a higher glycerophosphocholine to phosphocholine ratio. Moreover, the presence of somatic cells was found to affect milk quality. Sundekilde et al [25] classified BHBA, lactate, lactose, hippurate, acetate, butyrate, and fumarate in milk as metabolites associated with somatic cell production, wherein the concentrations of hippurate and fumarate decreased with increasing number of somatic cells [26]. In addition, choline, carnitine, citrate, and lactose in milk are known to be associated with coagulation properties [27], and citirate, N-acetylcarbohydrates, trimethylamine, lecithin, and lactose in milk are known to be closely associated with the milk quality [28–30]. All metabolites associated with somatic cells were identified in this study, except for lactate and hippurate; further, the metabolites associated with coagulation properties were also detected. In addition, all metabolites associated with milk quality were detected except N-acetylcarbohydrates and lecithin, and all the ketone body metabolites were observed. Therefore, milk quality testing using ^1^H-NMR spectroscopy might be a viable tool for detecting the metabolic disease of ketosis in dairy cattle.

## IMPLICATIONS

Proton nuclear magnetic resonance spectroscopy and statistical analyses were employed to analyze the metabolites in dairy cattle rumen fluid and milk. The metabolites measured in the rumen fluid and milk were mostly consistent with those reported in studies conducted abroad, and may be useful for predicting metabolic diseases, and milk quality. Furthermore, this report on metabolites in ruminant biofluids, which was achieved using the proton nuclear magnetic resonance analysis, will contribute to all the future ruminal metabolism studies in Korea.

## Figures and Tables

**Table 1:** Ingredients and nutrients of the experimental diets

Items	Value (% of dry matter)
Ingredients	
Concentrate	15.3
Soybean meal	2.40
Corn silage	47.2
Alfalfa hay	7.10
Tall fescue	9.40
Timothy	5.90
Energy booster1)	7.10
Cash gold1)	4.50
Lyzin-plus2)	0.20
Limestone	0.20
Zin care1)	0.10
Supex-F1)	0.50
Trace minerals3)	0.05
Vitamins premix4)	0.05
Chemical composition	
Dry matter (%)	53.2
Crude protein	10.0
Neutral detergent fiber	28.2
Acid detergent fiber	16.9
Calcium	0.40
Phosphorus	0.15

1) Cofavet, Cheonan, Korea.

2) A.N.Tech, Cheonan, Korea.

3) Trace minerals, contained 0.40% Mg, 0.20% K, 4.00% S, 0.08% Na, 0.03% Cl, 400 mg of Fe/kg, 60,042 mg of Zn/kg, 16,125 mg of Cu/kg, and 42,375 mg of Mn/kg.

4) Vitamins premix, provided approximately 5,000 KIU of vitamin A/kg, 1,000 KIU of vitamin D/kg, 33,500 mg of vitamin E/kg, and 2,400 mg of vitamin C/kg.

**Table 2:** Average concentrations (mean±standard deviation) of top 30 metabolites in rumen fluid by ^1^H-NMR analysis (n≥4)

Metabolites	Classification	Concentration (μM)
Acetate	Organic acids	14,474.47±1,900.36
Propionate	Organic acids	4,434.27±716.46
Butyrate	Organic acids	3,097.57±566.60
Valerate	Organic acids	330.00±61.46
Isobutyrate	Organic acids	165.76±23.15
Acetamide	Organic acids	118.95±19.22
Methylamine	Amines	115.93±22.24
3-Phenylpropionate	Others	108.53±19.83
Isovalerate	Organic acids	71.12±44.13
Glucose	Carbohydrates	65.08±22.81
Caprate	Lipids	60.98±19.87
Isopropanol	Alcohols	42.88±52.55
Ribose	Carbohydrates	36.33±15.15
2-Aminobutyrate	Amino acids	27.95±10.88
Alanine	Amino acids	26.75±14.31
Phenylacetate	Organic acids	22.53±12.09
Maltose	Carbohydrates	22.15±14.79
Glucose-6-phosphate	Carbohydrates	21.18±23.44
Alloisoleucine	Carboxylic acids	19.98±12.61
N-acetylglucosamine	Carbohydrates	19.07±17.91
3-Hydroxy-3-methylglutarate	Lipids	16.45±7.26
N-acetylglycine	Carboxylic acids	16.23±7.02
Xylitol	Carbohydrates	15.75±14.96
Biotin	Others	14.58±13.32
Lactose	Carbohydrates	11.23±6.37
Fructose	Carbohydrates	10.40±7.98
Erythritol	Carbohydrates	9.50±6.46
Uracil	Nucleosides, nucleotides	9.25±4.26
3-Hydroxyphenylacetate	Carboxylic acids	9.20±9.31
Imidazole	Imidazolinones	9.16±2.55

1 H-NMR, proton nuclear magnetic resonance.

**Table 3:** Average concentrations (mean±standard deviation) of top 30 metabolites in milk by ^1^H-NMR analysis (n≥4)

Metabolites	Classification	Concentration (μM)
Lactose	Carbohydrates	72,183.65±12,418.97
Guanidoacetate	Carboxylic acids	8,620.95±2,606.36
Ethylene glycol	Lipids	3,860.08±859.05
1,3-Dihydroxyacetone	Carbohydrates	2,953.88±2,719.11
Glycine	Amino acids	2,154.20±722.28
Glycolate	Lipids	1,701.72±984.03
Glycylproline	Carboxylic acids	348.80±259.79
Urea	Aliphatic acylic compounds	316.38±66.92
sn-Glycero-3-phosphocholine	Others	267.63±110.73
Arabinose	Others	263.78±362.78
Ribose	Carbohydrates	240.95±160.76
Isocitrate	Carbohydrates	226.58±157.30
Fructose	Carbohydrates	217.50±72.93
Creatine phosphate	Carboxylic acids	202.88±113.17
N-acetylglucosamine	Carbohydrates	201.57±42.18
Betaine	Others	186.20±182.74
Galactose	Carbohydrates	171.65±83.84
Lactulose	Carbohydrates	165.84±61.39
Choline	Lipids	150.60±140.69
Gluconate	Organic acids	121.65±80.61
Mannose	Carbohydrates	106.08±81.75
O-Phosphocholine	Aliphatic acylic compounds	102.15±142.06
Cellobiose	Others	100.14±40.66
Xylose	Carbohydrates	99.42±61.97
Trimethylamine N-oxide	Aliphatic acylic compounds	98.88±203.24
Fucose	Others	94.64±48.42
O-acetylcarnitine	Lipids	83.63±48.79
Acetone	Others	80.68±79.94
N-carbamoyl-β-alanine	Organic acids	76.58±58.47
Tartrate	Benzoic acids	72.53±29.92

1 H-NMR, proton nuclear magnetic resonance.

**Table 4:** Pathway analysis with common quantified (n≥4) metabolites in rumen fluid and milk

Metabolic pathway	Total Cmpd1)	Hits2)	p-value	−Log (p-value)	FDR3)	Impact4)
Pyruvate metabolism	22	1	3.42×10^−11^	24.10	3.42×10^−10^	0.06
Glycolysis/gluconeogenesis	26	1	3.42×10^−11^	24.10	3.42×10^−10^	0.03
Glyoxylate and dicarboxylate metabolism	32	1	3.42×10^−11^	24.10	3.42×10^−10^	0.00
Galactose metabolism	27	2	1.20×10^−8^	18.24	9.01×10^−8^	0.15
Glycerophospholipid metabolism	26	3	2.50×10^−5^	10.60	1.50×10^−4^	0.08
Ether lipid metabolism	20	1	5.47×10^−5^	9.81	2.73×10^−4^	0.00
Starch and sucrose metabolism	18	2	2.84×10^−4^	8.17	1.22×10^−3^	0.12
Tryptophan metabolism	41	2	6.14×10^−4^	7.40	2.30×10^−3^	0.05
Glycine, serine and threonine metabolism	34	3	7.44×10^−4^	7.20	2.42×10^−3^	0.13
Vitamin B6 metabolism	9	2	8.06×10^−4^	7.12	2.42×10^−3^	0.08
Arginine and proline metabolism	38	1	1.52×10^−3^	6.49	4.15×10^−3^	0.00
Amino sugar and nucleotide sugar metabolism	37	2	5.26×10^−3^	5.25	1.32×10^−2^	0.07
Histidine metabolism	16	2	9.23×10^−3^	4.69	2.13×10^−2^	0.24
Phenylalanine metabolism	12	1	1.51×10^−2^	4.19	3.24×10^−2^	0.00
Citrate cycle (tricarboxylic acid cycle)	20	1	2.32×10^−2^	3.76	4.09×10^−2^	0.03
Propanoate metabolism	28	1	2.32×10^−2^	3.76	4.09×10^−2^	0.00

1) Total Cmpd, the total number of compounds in the pathway.

2) Hit, the actually matched number from the user uploaded data.

3) FDR, the p-value adjusted using false discovery rate.

4) Impact, the pathway impact value calculated from pathway topology analysis.
