# Supplementary material for: Metabolomics comparison of rumen fluid and milk in dairy cattle using proton nuclear magnetic resonance spectroscopy
Source: Anim Biosci. 2020 Jun 24;34(2):213–22. doi: 10.5713/ajas.20.0197 (PMC7876715; doi:10.5713/ajas.20.0197)
Supplement: Supplementary file 1 [file ajas-20-0197-suppl.pdf]

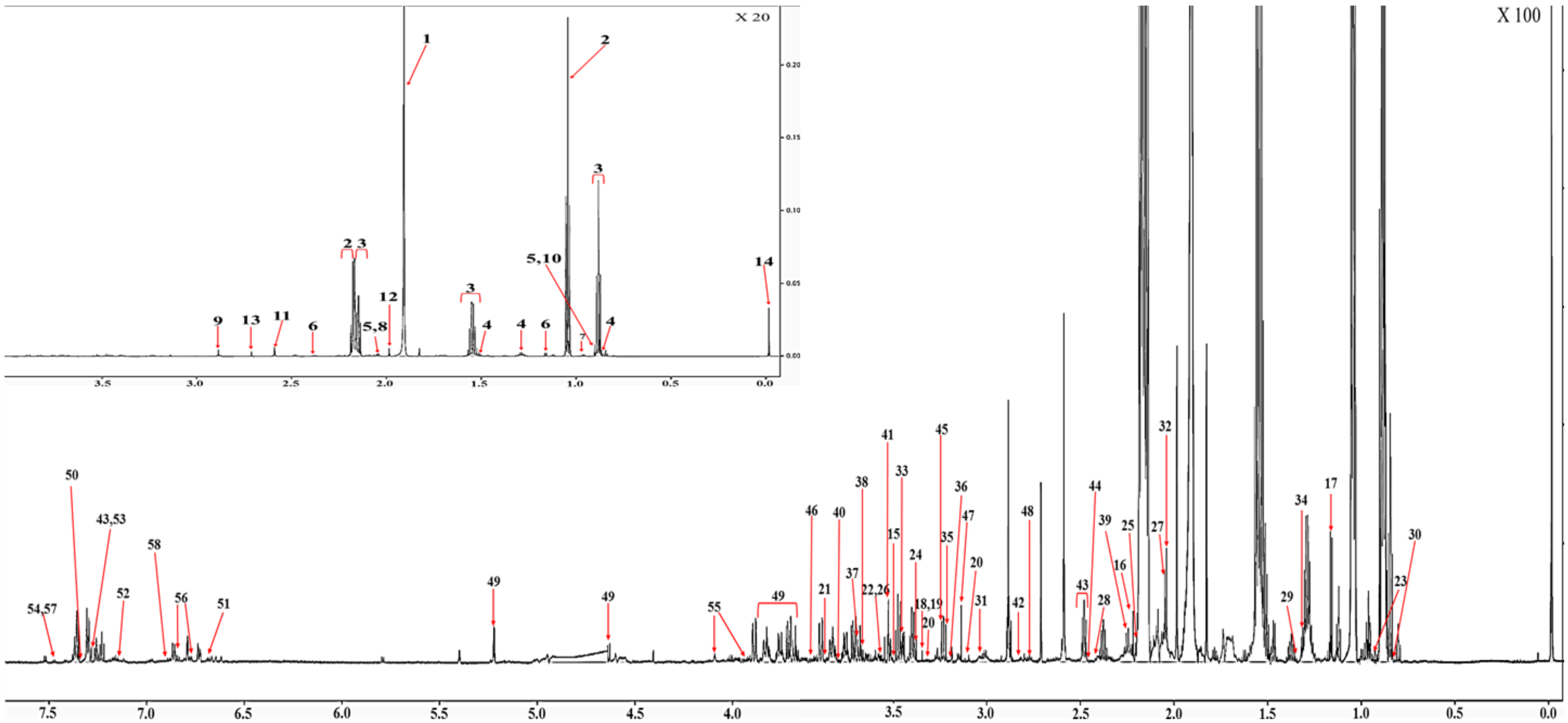

Supplementary Figure 1. Representative 800 MHz ^{1}H-NMR spectra (0.0 ~ 10.0 ppm) of rumen fluid samples.

1, Acetate; 2, Propionate; 3, Butyrate; 4, Valerate; 5, Isovalerate; 6, 3-Hydroxybutyrate; 7, 2-Aminobutyrate; 8, N-Acetylglycine; 9, Trimethylamine; 10, Pantothenate; 11, Methylamine; 12, Acetamide; 13, Dimethylamine; 14, TSP; 15, Acetoacetate; 16, Acetone; 17, Isopropanol; 18, Methanol; 19, O-Phosphocholine; 20, Histamine; 21, Anserine; 22, Glycine; 23, Leucine; 24, 3,4-Dihydroxybenzeneacetate; 25, o-Cresol; 26, 1,3-Dihydroxyacetone; 27, N-Acetylglucosamine; 28, Succinate; 29, 2-Hydroxyisobutyrate; 30, 2-Hydroxyisovalerate; 31, Creatine phosphate; 32, N-Acetylglycine; 33, trans-Aconitate; 34, 3-Hydroxy-3-methylglutarate; 35, Carnitine; 36, Choline; 37, Ethylene glycol; 38, Glutarate monomethyl ester; 39, Thymol; 40, N-Nitrosodimethylamine; 41, Phenylacetate; 42, Succinylacetone; 43, 3-Phenylpropionate; 44, 4-Pyridoxate; 45, Betaine; 46, Caffeine; 47, Dimethyl sulfone; 48, Levulinate; 49, Glucose; 50, N-Phenylacetylglycine; 51, Homogentisate; 52, p-Cresol; 53, Syringate; 54, Vanillate; 55, Ribose; 56, 3-Hydroxyphenylacetate; 57, Imidazole; 58, 2-Hydroxyphenylacetate.

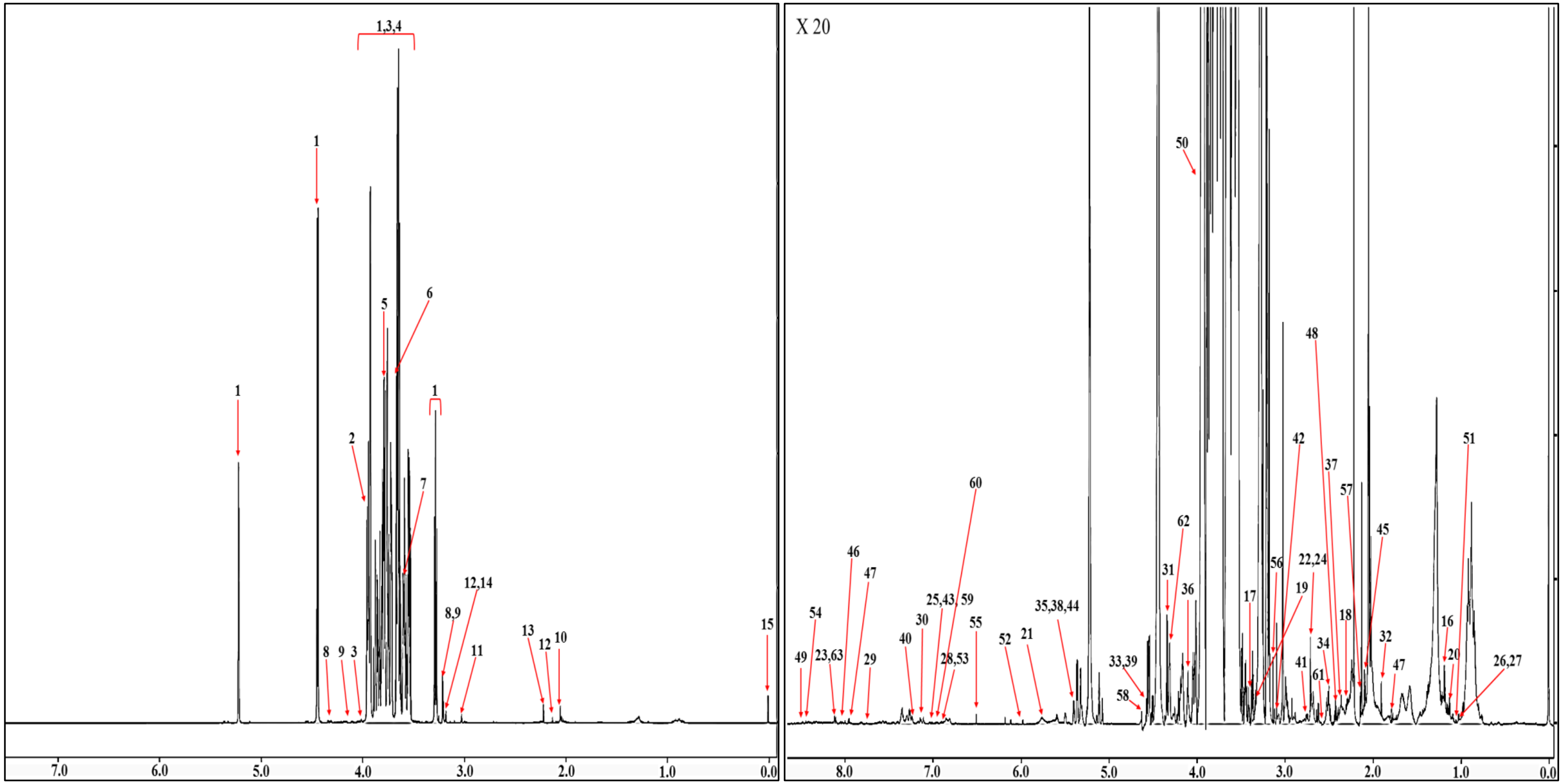

Supplementary Figure 2. Representative 800 MHz ^{1}H-NMR spectra (0.0 ~ 10.0 ppm) of milk samples.

1, Lactose; 2, Threonate; 3, Galactitol; 4, Glucitol; 5, Guanidoacetate; 6, Ethylene glycol; 7, Glycine; 8, sn-Glycero-3-phosphocholine; 9, O-Phosphocholine; 10, N-Acetylglucosamine; 11, Creatine phosphate; 12, O-Acetylcarnitine; 13, Acetone; 14, Choline; 15, TSP; 16, 3-Hydroxybutyrate; 17, Acetoacetate; 18, Acetone; 19, Methanol; 20, Propylene glycol; 21, Urea; 22, Dimethylamine; 23, Histamine; 24, Sarcosine; 25, Anserine; 26, Isoleucine; 27, Valine; 28, 4-Hydroxy-3-methoxymandelate; 29, Acetylsalicylate; 30, o-Cresol; 31, Tartrate; 32, Acetate; 33, Galactose; 34, Isocitrate; 35, Maltose; 36, Ribose; 37, Succinate; 38, Sucrose; 39, Xylose; 40, 3-Hydroxyphenylacetate; 41, 5-Aminolevulinate; 42, cis-Aconitate; 43, Homovanillate; 44, Maltose; 45, N-Acetylcysteine; 46, N-Acetylglucosamine; 47, Nα-Acetyllysine; 48, Carnitine; 49, Imidazole; 50, Glycolate; 51, Methylsuccinate; 52, UDP-N-Acetylglucosamine; 53, 3-Hydroxymandelate; 54, Formate; 55, Fumarate; 56, N-Nitrosodimetylamine; 57, O-Acetylcholine; 58, Cellobiose; 59, N-Acetylserotonin; 60, Pyridoxine; 61, Riboflavin; 62, sn-Glycero-3-phosphocholine; 63, Theophylin.

Supplementary Table 1. Measured metabolites concentration in rumen fluid and milk samples by ^{1}H-NMR analysis (Mean ± Standard Deviation, n≥2)

Metabolites (μM)	Rumen fluid	Milk
Alcohols		
Isopropanol*	42.88 ± 52.55	21.03 ± 14.34
Methanol*	3.43 ± 1.67	10.65 ± 6.86
Propylene glycol	3.03 ± 2.22	16.85 ± 13.58
Aliphatic acylic compounds		
O-Phosphocholine*	1.62 ± 0.98	102.15 ± 142.06
Trimethylamine N-oxide	1.60 ± 1.11	98.88 ± 203.24
Urea	ND	316.38 ± 66.92
Amines		
Dimethylamine	17.86 ± 14.96	12.30 ± 9.82
Histamine*	2.15 ± 1.74	4.06 ± 1.65
Kynurenine	4.43 ± 2.21	13.40 ± 3.90
Methylamine*	115.93 ± 22.24	8.68 ± 10.82
Sarcosine*	1.65 ± 1.92	8.50 ± 3.44
Trimethylamine	8.53 ± 11.49	1.00 ± 0.67
Amino acids		
2-Aminobutyrate	27.95 ± 10.88	ND
2-Furoylglycine	ND	2.86 ± 0.75
3-Aminoisobutyrate	11.16 ± 11.20	ND
Anserine*	5.31 ± 4.56	13.30 ± 13.92
Creatine	ND	54.45 ± 18.51
N-Phenylacetylglycine	4.65 ± 1.72	ND
γ-Glutamylphenylalanine	ND	21.20 ± 6.08
π-Methylhistidine	4.93 ± 2.25	ND
Alanine	26.75 ± 14.31	6.56 ± 3.56
Glycine	ND	2154.20 ± 722.28
Isoleucine	7.83 ± 3.00	9.10 ± 7.77
Leucine	5.6 ± 1.31	ND
Methionine*	5.78 ± 6.70	5.86 ± 3.64
Valine	ND	5.98 ± 5.58
Benzoic acids		
3,4-Dihydroxybenzeneacetate	5.88 ± 2.30	ND
3-Hydroxymandelate	ND	5.02 ± 1.77
4-Hydroxy-3-methoxymandelate*	2.40 ± 2.23	3.21 ± 1.07

Metabolites (μM)	Rumen fluid	Milk
Benzoic acids		
4-Hydroxyphenylacetate*	5.02 ± 2.49	7.78 ± 4.42
Acetylsalicylate	2.16 ± 1.12	3.72 ± 1.61
Homogentisate	4.56 ± 1.24	ND
Isoeugenol	ND	2.90 ± 2.27
Mandelate	1.90 ± 0.26	2.50 ± 0.73
o-Cresol*	8.30 ± 2.79	7.36 ± 4.84
p-Cresol	7.94 ± 1.64	ND
Salicylurate	ND	4.35 ± 1.11
Syringate	0.80 ± 0.36	ND
Tartrate	ND	72.53 ± 29.92
Vanillate	ND	1.76 ± 0.30
Carbohydrates		
1,3-Dihydroxyacetone*	2.97 ± 1.66	2953.88 ± 2719.11
Acetoacetate*	8.70 ± 8.47	49.06 ± 16.04
Arabinitol	ND	2127.10 ± 927.02
Erythritol	9.50 ± 6.45	ND
Fructose*	10.40 ± 7.97	217.50 ± 72.93
Galactitol	ND	4565.77 ± 3902.48
Galactonate	ND	54.45 ± 18.51
Galactose	ND	171.65 ± 83.84
Glucitol	ND	3880.23 ± 1173.63
Glucose	65.08 ± 22.81	ND
Glucose-6-phosphate	21.17 ± 23.44	ND
Glutathione	20.00 ± 3.14	ND
Isocitrate	40.80 ± 28.83	226.58 ± 157.30
Lactose*	11.23 ± 6.37	72183.65 ± 12418.97
Lactulose	8.63 ± 2.30	165.84 ± 61.39
Maltose*	22.15 ± 14.79	49.75 ± 20.33
Mannose	ND	106.08 ± 81.75
N-Acetylglucosamine*	19.06 ± 17.90	201.57 ± 42.18
Pyruvate	12.3 ± 1.34	ND
Ribose*	36.32 ± 15.15	240.95 ± 160.76
Succinate*	5.34 ± 2.01	3.00 ± 0.70

Supplementary Table 2. *Continued*

Metabolites (μM)	Rumen fluid	Milk	Metabolites (μM)	Rumen fluid	Milk
Carbohydrates			***Imidazolinones***		
Sucrose*	3.35 ± 2.54	54.78 ± 17.12	Creatinine	ND	72.13 ± 23.49
Trehalose	ND	26.88 ± 21.42	Imidazole*	9.16 ± 2.55	8.00 ± 4.06
Xylitol	15.75 ± 14.95	ND	***Indoles***		
Xylose	ND	99.42 ± 61.97	5-Hydroxyindole-3-acetate*	2.46 ± 1.56	1.75 ± 0.48
Carboxylic acids			***Lipids***		
2-Hydroxyisobutyrate	2.82 ± 1.38	ND	2-Ethylacrylate	ND	3.80 ± 2.57
3-Hydroxyisobutyrate	ND	4.10 ± 2.52	2-Methylglutarate	ND	3.06 ± 0.75
3-Hydroxyisovalerate	4.93 ± 3.52	18.26 ± 5.22	3,5-Dibromotyrosine	8.70 ± 9.46	1.26 ± 0.05
3-Hydroxyphenylacetate*	9.20 ± 9.31	20.40 ± 5.96	3-Hydroxy-3-methylglutarate*	16.45 ± 7.25	12.05 ± 11.40
5-Aminolevulinate	1.66 ± 0.75	20.60 ± 16.21	3-Hydroxybutyrate*	6.08 ± 4.03	52.63 ± 33.92
Alloisoleucine	19.97 ± 12.61	ND	3-Methylglutarate	ND	59.90 ± 55.77
cis-Aconitate	ND	8.45 ± 4.40	Caprate	60.98 ± 19.86	ND
Creatine phosphate*	1.48 ± 0.74	202.88 ± 113.17	Caprylate	147.23 ± 36.12	ND
Glycylproline	ND	348.80 ± 259.79	Carnitine	ND	25.04 ± 29.22
Guanidinosuccinate	ND	45.03 ± 2.74	Choline*	1.18 ± 0.82	150.60 ± 140.69
Guanidoacetate	ND	8.62 ± 2.60	Ethylene glycol	10.46 ± 8.99	3860.08 ± 2606.36
Homovanillate*	2.35 ± 2.34	2.28 ± 0.58	Glutaric acid monomethyl ester	9.66 ± 4.27	ND
Hydroxyacetone	ND	2.70 ± 1.45	Glycolate	ND	1701.72 ± 984.03
Maleate	ND	1.76 ± 0.96	Methylsuccinate	ND	3.74 ± 0.95
Malonate*	2.66 ± 0.78	11.85 ± 6.43	O-Acetylcarnitine	ND	83.63 ± 48.78
N,N-Dimethylformamide	0.73 ± 0.23	4.42 ± 2.73	Thymol	4.88 ± 1.57	ND
N-Acetylaspartate	5.65 ± 3.42	19.96 ± 12.65	***Nucleosides, Nucleotides***		
N-Acetylcysteine	ND	25.06 ± 6.70	GTP	ND	2.83 ± 0.32
N-Acetylglutamine	ND	32.15 ± 14.48	UDP-N-Acetylglucosamine	ND	1.38 ± 0.29
N-Acetylglycine	16.23 ± 7.01	ND	Uracil	9.25 ± 4.26	ND
N-Acetyltyrosine	ND	6.50 ± 1.64	***Organic acids***		
N-Carbamoylaspartate	ND	76.57 ± 58.47	2-Oxoglutarate	ND	45.10 ± 6.03
N-Phenylacetylphenylalanine	ND	8.82 ± 2.39	3-Hydroxykynurenine	ND	5.45 ± 1.13
Nα-Acetyllysine	8.26 ± 4.67	14.74 ± 7.19	Acetamide	118.95 ± 19.22	ND
Pantothenate*	3.72 ± 3.96	15.25 ± 17.71	Acetate*	14474.77 ± 1990.36	28.08 ± 15.54
Saccharopine	ND	47.83 ± 24.70	Butyrate	3097.57 ± 566.60	ND
trans-Aconitate	2.78 ± 2.55	ND	Ferulate*	1.77 ± 1.50	1.76 ± 1.18
Imidazolinones			Formate	ND	7.78 ± 2.04
Allantoin	10.00 ± 1.47	17.15 ± 5.96	Fumarate	ND	6.65 ± 2.02

Supplementary Table 3. *Continued*

Metabolites (μM)	Rumen fluid	Milk
Organic acids		
Gluconate	5.30 ± 1.70	121.65 ± 80.61
Isobutyrate	165.76 ± 23.15	ND
Isovalerate	71.11 ± 44.12	ND
Malate	16.43 ± 8.57	ND
N-Nitrosodimethylamine	6.06 ± 2.17	55.06 ± 78.57
O-Acetylcholine	ND	32.95 ± 53.06
Phenylacetate	22.53 ± 12.08	ND
Propionate	4434.27 ± 716.46	ND
Succinylacetone	6.38 ± 4.09	ND
Valerate	330.00 ± 61.46	ND
Others		
1,3-Dimethylurate	0.90 ± 0.62	7.50 ± 5.06
1,7-Dimethylxanthine	ND	5.85 ± 3.03
2-Hydroxyphenylacetate*	6.31 ± 2.47	7.76 ± 1.92
3-Methylxanthine*	0.71 ± 0.27	1.48 ± 0.71
3-Phenylpropionate	108.53 ± 19.83	30.80 ± 27.93
4-Pyridoxate*	2.05 ± 1.01	2.41 ± 2.22
Acetoin	4.80 ± 2.77	43.44 ± 9.51
Acetone*	8.36 ± 5.09	80.68 ± 79.94
Arabinose	ND	263.78 ± 362.78
Betaine*	3.83 ± 6.09	186.20 ± 182.76

Metabolites (μM)	Rumen fluid	Milk
Others		
Biotin*	14.57 ± 13.32	28.87 ± 20.30
Butanone	ND	2.37 ± 0.35
Caffeine*	1.85 ± 1.45	4.14 ± 3.08
Cellobiose	ND	100.14 ± 40.66
Desaminotyrosine	ND	6.06 ± 2.85
Dimethyl sulfone*	3.94 ± 1.93	13.13 ± 4.04
Fucose	10.56 ± 3.05	94.64 ± 48.41
Galactarate	ND	17.83 ± 9.56
Ibuprofen	ND	4.10 ± 1.70
Indole-3-acetate	3.06 ± 0.89	ND
Levulinate	7.20 ± 4.00	ND
Melatonin	ND	2.42 ± 0.72
Methylguanidine	1.60 ± 0.52	ND
N-Acetylserotonin*	1.36 ± 0.42	2.26 ± 1.26
N-Methylhydantoin	ND	3.61 ± 3.79
Pyridoxine*	0.76 ± 0.15	1.26 ± 0.53
Riboflavin	ND	1.38 ± 0.14
sn-Glycero-3-phosphocholine*	1.67 ± 0.98	267.63 ± 110.73
Theophylline	0.83 ± 0.51	2.33 ± 3.00
τ-Methylhistidine	ND	2.82 ± 1.43

^{1}H-NMR, proton nuclear magnetic resonance; ND, Not detected.

* Common metabolites were quantified in the rumen fluid and milk (n≥4)

Highlighted area quantified metabolites in the rumen fluid (n≥4)

Highlighted area quantified metabolites in milk (n≥4)
